# Oncogenomics of c-Myc transgenic mice reveal novel regulators of extracellular signaling, angiogenesis and invasion with clinical significance for human lung adenocarcinoma

**DOI:** 10.18632/oncotarget.21981

**Published:** 2017-10-23

**Authors:** Yari Ciribilli, Jürgen Borlak

**Affiliations:** ^1^ Centre for Integrative Biology (CIBIO), University of Trento, 38123 Povo (TN), Italy; ^2^ Centre for Pharmacology and Toxicology, Hannover Medical School, 30625 Hannover, Germany

**Keywords:** c-Myc transgenic mouse model of lung cancer, papillary adenocarcinomas, whole genome scans, c-Myc regulatory gene networks, c-Myc targeted regulators of extracellular signaling

## Abstract

The c-Myc transcription factor is frequently deregulated in cancers. To search for disease diagnostic and druggable targets a transgenic lung cancer disease model was investigated. Oncogenomics identified c-Myc target genes in lung tumors. These were validated by RT-PCR, Western Blotting, EMSA assays and ChIP-seq data retrieved from public sources. Gene reporter and ChIP assays verified functional importance of c-Myc binding sites. The clinical significance was established by RT-qPCR in tumor and matched healthy control tissues, by RNA-seq data retrieved from the TCGA Consortium and by immunohistochemistry recovered from the Human Protein Atlas repository. In transgenic lung tumors 25 novel candidate genes were identified. These code for growth factors, Wnt/β-catenin and inhibitors of death receptors signaling, adhesion and cytoskeleton dynamics, invasion and angiogenesis. For 10 proteins over-expression was confirmed by IHC thus demonstrating their druggability. Moreover, c-Myc over-expression caused complete gene silencing of 12 candidate genes, including *Bmp6, Fbln1* and *Ptprb* to influence lung morphogenesis, invasiveness and cell signaling events. Conversely, among the 75 repressed genes TNFα and TGF-β pathways as well as negative regulators of IGF1 and MAPK signaling were affected. Additionally, anti-angiogenic, anti-invasive, adhesion and extracellular matrix remodeling and growth suppressive functions were repressed. For 15 candidate genes c-Myc-dependent DNA binding and transcriptional responses in human lung cancer samples were confirmed. Finally, Kaplan–Meier survival statistics revealed clinical significance for 59 out of 100 candidate genes, thus confirming their prognostic value.

In conclusion, previously unknown c-Myc target genes in lung cancer were identified to enable the development of mechanism-based therapies.

## INTRODUCTION

The c-Myc transcription factor is an essential component of the cell cycle machinery and regulates cell proliferation and growth in response to mitogenic stimuli [1]. Given its hyperactivity in diverse malignancies, c-Myc has emerged as an important drug target [2, 3]. Although the exact mechanism by which c-Myc causes cellular transformation remains unclear, inhibition of c-Myc activity was shown to be of therapeutic benefit in different cancer models including KRas-driven lung cancer in mice [4].

To better understand the gene regulatory networks linked to oncogenic activation of c-Myc in lung adenocarcinomas a transgenic disease model was developed whereby c-Myc is targeted to respiratory epithelium by use of a gene construct that contains regulatory elements of the surfactant protein C (SPC) promoter [5]. This allowed an assessment of molecular events associated with c-Myc hyperactivity *in vivo*. We recently reported whole genome scans of well-defined transgenic lung adenocarcinomas to better define the genetic program induced by c-Myc over-expression in lung epithelial cells leading to neoplasia [6, 7]. This research identified 162 and 301 genes as up- and down-regulated. The genes were further analyzed with regard to their biological functions, known responsiveness to c-Myc and the presence of c-Myc binding sites in their promoters (putative c-Myc targets). The coded biological functions of regulated genes informed on a broad spectrum of pivotal metabolic and signaling pathways as regulated in lung cancer. In our concluding study we now report c-Myc's ability to modulate extracellular signaling and to endorse cell proliferation by altering cytoskeleton dynamics, cell adhesion, invasiveness and angiogenic capacities.

## RESULTS

The solid tumors of c-Myc transgenic mice were classified as papillary lung adenocarcinomas [6]. To follow gene expression changes during disease progression RNA was prepared from small, middle and large sized tumors and analyzed by whole genome microarrays. Note, with the exception of middle sized tumors, i.e. 5 mm in diameter, the small (1 mm) and large tumors (> 10 mm) expressed *c-Myc* transcripts > 50-fold. Given in Table 1 are the Fold Changes (FC) of a panel of genes that were either up- or down-regulated upon c-Myc over-expression in PLACs and that were further analyzed in this study (for an extended presentation of the whole list of differentially expressed genes refer to Supplementary Table 1). Statistical analysis of replicates with *T*-test and stringent comparative ranking was performed to identify significantly regulated genes in tumors as compared to non-transgenic healthy lungs. The thresholds for significance testing were defined as mean FC > 3, *p*-value in *T*-test < 0.05, and 100% “Increase” calls in comparative ranking analysis at least in one set of tumors for up-regulated genes, and FC < -3, *p*-value in *T*-test < 0.05, and 100% “Decrease” calls for down-regulated genes. Significantly regulated genes were further analyzed with regard to their functional annotation and previous knowledge on c-Myc responsiveness, based on publically available information, and for the presence of c-Myc binding sites in their promoters using positional weight matrices. The data analysis focused on extracellular signaling, adhesion, angiogenesis and invasion, and Supplementary Table 1 compiles genes categorized into functional groups and their known c-Myc responsiveness. Representative examples include the autocrine growth factor amphiregulin and the mitogen-activated protein kinase kinase 1 (MEK) both of which were specifically up-regulated, whereas genes negatively influenced by Erk signaling, like phosphatase Dusp1, Ptpre and Rassf5, i.e. a pro-apoptotic Ras-effector, were repressed. Moreover, increased expression of the GTPase Ras homolog family member U (Rhou) and the WNT signaling pathway regulator Frat2 was observed, though the expression of the T-cell-specific transcription factor 1 (Tcf7) and Sox7 with defined roles in Wnt/beta-catenin signaling and cell fate was repressed. In lung tumors up-regulation of Tnf receptor-associated factor 4 and reduced expression of the death receptor protein Fas was observed to repress cell death receptor signaling. Remarkably, amongst the many transcripts repressed in lung tumors were those coding for growth-inhibition and included *Igfbp4* to 6 and *Nfkbia,* the latter inhibiting NFκB. Their reduced expression sustains excessive survival signaling via the IGF receptor and in such a way suppresses the apoptotic activity of c-Myc.

**Table 1:** A selection of significantly regulated genes in c-Myc-induced lung papillary adenocarcinoma^*^

ACC	Gene Symbol	Gene Title	Fold Change		
			Size of tumors		
			Small	Middle	Large
**(A) Up-regulated genes in c-Myc induced lung tumors**					
L00039	Myc	Myelocytomatosis oncogene	51.0	23.4	58.6
AI467390	Map2k1	Mitogen activated protein kinase kinase 1	2.7	2.7	3.6
U15443	Ros1	Ros1 proto-oncogene	9.3	13.3	21.9
AV109962	Traf4	Tnf receptor associated factor 4	4.3	5.0	4.5
X63099	Gjb3	Gap junction membrane channel protein beta 3	8.7	7.5	8.8
M22832	Krt18	Keratin complex 1. acidic. gene 18	2.9	3.2	3.6
AF011450	Col15a1	Procollagen. type XV	1.7	5.1	4.6
AA170696	Icam1	Intercellular adhesion molecule	3.3	1.8	2.8
AW228162	Dsc2	Desmocollin 2	4.1	4.1	4.4
X81627	Lcn2	Lipocalin 2	4.8	7.3	4.7
AI786089	Kng	Kininogen	22.4	37.1	12.6
AI042655	Hgfac	Hepatocyte growth factor activator	4.7	9.6	8.7
AA608277	Adora2b	Adenosine A2b receptor	5.8	4.1	2.4
AW230369	Spint1	Serine protease inhibitor. Kunitz type 1	3.0	2.7	2.3
AA726223	Adam19	A disintegrin and metalloproteinase domain 19 (meltrin beta)	3.2	3.3	3.1
AW047185	Thop1	Thimet oligopeptidase 1	4.0	2.8	4.2
M83649	Fas	Tumor necrosis factor receptor superfamily. member 6	–4.2	–2.6	–3.6
AI850533	Bmp6	Bone morphogenetic protein 6	–15.5	–34.8	–22.8
AA717826	Dpt	Dermatopontin	–5.0	–6.7	–16.6
L48015	Acvrl1	Activin A receptor. type II-like 1	–5.9	–6.7	–7.6
L12447	Igfbp5	Insulin-like growth factor binding protein 5	–6.5	–1.9	–4.9
X81584	Igfbp6	Insulin-like growth factor binding protein 6	–8.8	–8.8	–18.9
AB020886	Akap12	A kinase (PRKA) anchor protein (gravin) 12	–5.8	–9.0	–11.9
AI747654	Cav1	Caveolin-1. caveolae protein	–4.0	–5.6	–6.5
AF080090	Sema3f	Sema domain. immunoglobulin domain (Ig). short basic domain. secreted. (semaphorin) 3F	–4.9	–5.4	–5.6
X70854	Fbln1	Fibulin 1	–3.8	–3.1	–10.1
AI853217	Cdh5	Cadherin 5	–7.2	–9.9	–10.0
X14432	Thbd	Thrombomodulin	–16.0	–27.1	–55.8
X65493	Icam2	Intercellular adhesion molecule 2	–12.0	–30.6	–48.1
X71426	Tek	Endothelial-specific receptor tyrosine kinase	–12.6	–16.9	–26.3
AI843063	Vwf	Von Willebrand factor homolog	–8.4	–7.7	–16.9
U30292	Col13a1	Procollagen. type XIII. alpha 1	–6.2	–7.3	–16.7
X79199	Clec3b	Tetranectin (plasminogen binding protein)	–11.1	–11.1	–11.1
U82758	Cldn5	Claudin 5	–9.1	–17.8	–45.9
Z38110	Pmp22	Peripheral myelin protein	–6.2	–9.6	–16.3
X58289	Ptprb	Protein tyrosine phosphatase. receptor type. B	–14.3	–21.3	–40.9
D76440	Ndn	Necdin	–7.6	–8.4	–10.4
Z16406	Meox2	Mesenchyme homeobox 2	–8.6	–12.6	–12.6

^*^ = A comprehensive list of differentially expressed genes is given in Supplementary Table 1.

Furthermore, expression of genes involved in anti-growth TGF-β signaling such as Bmp6 and TGF-β1 as well as Acvrl1, a member of the TGF-β receptor family and the tumor suppressor Gadd45β was reduced. Likewise, inhibitors of cell proliferation, i.e. Gas3, Ndrg2, Lox, Meox2, Ptprb and Ptprg, were repressed as they inhibit transformation, such as Lats2 and the scaffolding proteins Akap 12 and caveolin-1.

In lung tumors proteins involved in cell-cell and cell-ECM junctions, i.e. laminin alpha 3, integrin alpha 8, cadherin 5, protocadherin alpha 4 and 6 were repressed, but the oncogenic transcription factor Ect2 was up-regulated. Moreover, down-regulation of Vsnl1 and calpain2 in lung tumors evidenced c-Myc's ability to modulate cytoskeleton dynamics in transformed cells.

Increased expression of several proteases like HGF activator, Adam19, lipocalin, hepsin and thimet oligopeptidase 1 was observed, but down-regulation of Reck, visinine-like1 and semaphorins may well contribute to invasion and angiogenesis.

Some of the differentially regulated genes were uniquely expressed in tumors and coded for extracellular proteins, i.e. HGF activator, thimet oligopeptidase, and kininogen, which defines them as potential tumor marker candidates and play a critical role in tissue repair and intracellular signaling. Conversely, expression of protocadherin alpha 4 and 6, Lama3, procollagen XIIIalpha1, dermatopontin and claudin5 was uniquely expressed in healthy lung, as was the expression of Bmp6, Ndrg2, Igfbp4, Sema3f and fibulin1. Silencing of these genes in respiratory epithelium may be an essential step towards malignant transformation. These genes are highlighted in Table 1A (up-regulated genes) and 1B (down-regulated genes) and similarly in Supplementary Table 1A and 1B.

Quantitative differences in gene expression between small- and large-size tumors included 2- to 3-fold changes in expression levels. With small-sized tumors expression of adenosine A2b receptor and of kininogen was increased to foster angiogenic signaling and to inhibit ERK activity, whereas expression of the proto-oncogene Ros1, the protease Hgfac, procollagen type XV, and claudin2 were significantly increased in large tumors. Moreover, inhibitor of growth, i.e. Tgfb1, Gadd45b, Igfbp6, A-kinase-anchoring protein (Akap12) and protein tyrosine phosphatase, non-receptor type substrate 1 were highly significantly repressed, from which their important role in tumor progression can be inferred.

Among significantly regulated genes were also the known c-Myc-responsive genes Tnfrsf6 and Hoxa5, which are part of the Tnf-signaling cascade, the scaffolding proteins Akap12 and Cav1, the phosphatase Dusp1 and Lox as well as the established c-Myc targets mucin1 and Icam1 which were up-regulated in lung tumors. Conversely, Icam2 expression was almost complete silenced in lung tumors, and Tcf7 and Notch4 were likewise significantly repressed in expression (Table 1 and Supplementary Table 1).

### RT-PCR and Western blotting of transgenic tumor regulated genes

**Figure 1:**
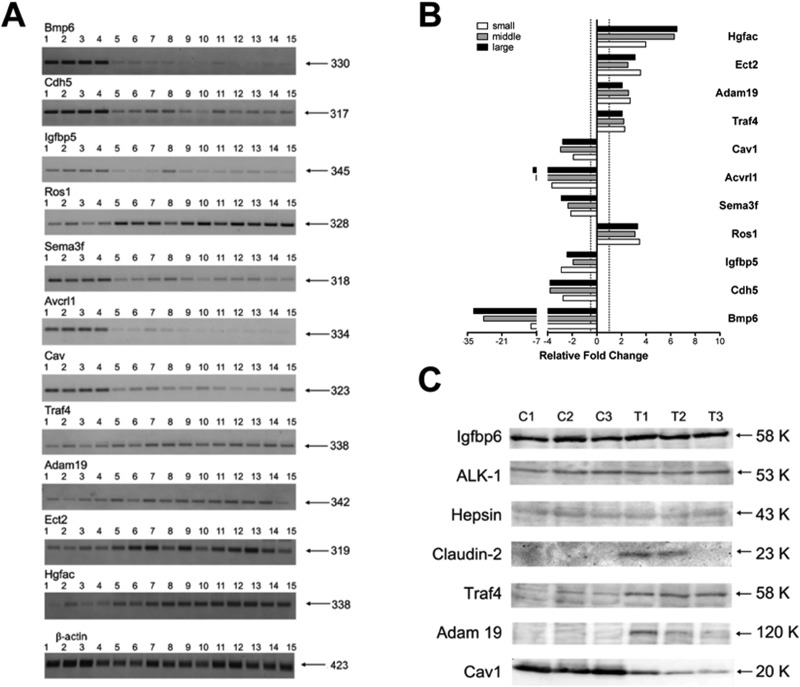
RT-PCR and Western blotting of novel c-Myc candidate genes in papillary lung adenocarcinomas (**A**) Semi-quantitative RT-PCRs for selected genes. Lanes 1–4: control lung tissues; lanes 5–8: pools of small-size tumors; lanes 9–11: middle-size tumors; lanes 12–15: large-size tumors. Indicated on the right is the size in base pairs of the PCR amplicons. (**B**) Bars represent the averages and standard deviations of band intensities from panel A for the three groups of samples (small, middle and large tumors) normalized on the β-actin reference gene. The area from each band was calculated with Image J software and used for generating averages. (**C**) Western blot analysis of selected genes. C1–C3: control non-transgenic lung; T1–T3: lung adenocarcinomas of SPC/c-Myc-transgenic mice. Indicated on the right is the size in kDa of immunoreactive bands.

RT-PCR of 5 up-regulated (Ros1, Traf4, Adam19, Ect2 and Hgfac) and 6 repressed (Bmp6, Cdh5, Igfbp5, Sema3f, Avcrl1 and Cav1) genes in 11 individual lung tumors and 4 control lungs demonstrated strong agreement between the two methods employed (Figure 1A–1B and Supplementary Table 2) while Western blotting (Figure 1C) provided additional evidence for regulation of the genes at the protein level, most notably for Claudin2, Traf4 and Adam19. Nonetheless, the proteins Hepsin, Alk1 (Acvrl1) and Igfbp6 were equally expressed in tumors and control lungs.

### *In silico* mapping of c-Myc binding sites in differentially expressed genes

Bioinformatics was applied to search for c-Myc binding sites in genomic sequences of regulated genes to provide evidence for c-Myc transcriptional control of newly identified gene candidates such as Rhou, involved in Wnt-signaling, Traf4, with anti-apoptotic function, Adam19, a membrane-anchored peptidase, Acvrl1, and Rbp1, with anti-proliferative functions. Similarly, connexins (Gjb3 and Gja1) and semaphorins (Sema3c and Sema7a) were identified as putative c-Myc target genes which belong to a previously unsuspected class of c-Myc-responsive genes. A summary of the data is given in Supplementary Table 1.

### EMSA assays to determine c-Myc DNA binding activity at gene specific promoters

**Figure 2:**
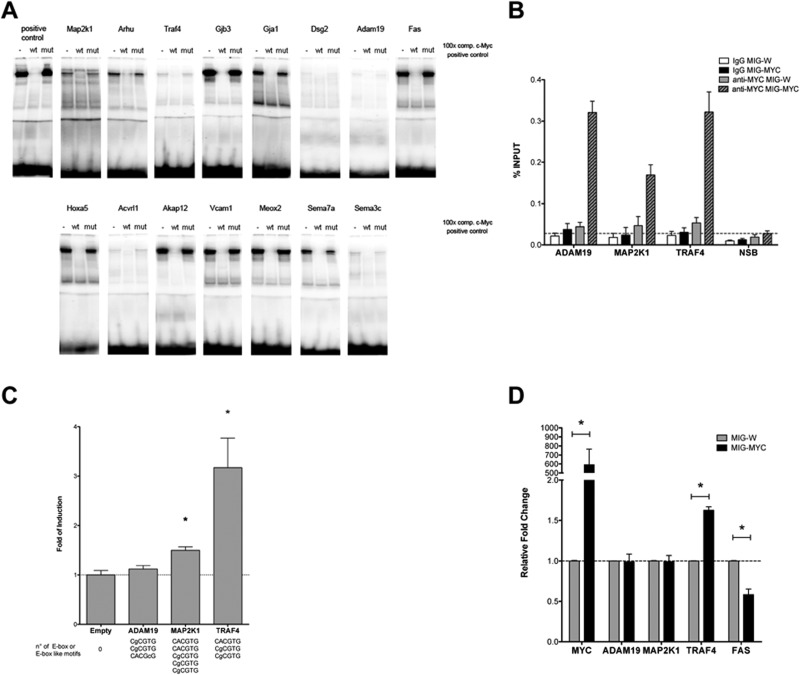
DNA binding activity of nuclear proteins to newly identified c-Myc target genes. (A) A total of 15 c-Myc binding sites were studied by EMSA band shift assay. We tested several commercially available C- and N-terminus directed antibodies to supershift the c-Myc DNA-protein complex. Unfortunately, none of the tested antibodies proved useful. Instead, we employed an established positive control and wild type and mutated competitor probes (100-fold excess) to assess specificity (see also Supplementary Table 1 for sequences of the oligonucleotide probes used for EMSA). **(B)** DNA binding ability was also studied through Chromatin IP on HEK 293T cells transiently transfected with an empty vector or a c-MYC over-expressing vector. qPCR measurements of selected genes were analyzed on immunoprecipitated DNA with an anti-MYC monoclonal antibody (clear and patterned gray bars) or mouse normal IgG (white and black bars). The non-specific binding of c-Myc over-expression is shown as a dashed line with qPCR values obtained for the ACTB promoter and exon 9 locus of CCNB1. The results are given as % of enrichment with respect to input sample, and bars represent the averages and standard deviations of two biological replicates. **(C)** Gene reporter assays to determine c-Myc responsiveness of Adam19, Map2k1 and Traf4 constructs. Dual luciferase assays were conducted in HEK 293T cells using the pCZ-REN-P-LUC retroviral vectors containing 2 kb regulatory regions of the selected genes. The number and sequences of E-box or E-box-like motifs are indicated beneath the bars. The reporter vector also contains the control Renilla luciferase to normalize for infection/transfection efficiency. A control vector or the MIG-MYC c-Myc expression vector was also co-transfected. Given are the average ratios of the fold of reporter induction obtained in cells with ectopic over-expression of c-Myc compared to control cells with endogenous c-Myc expression. Error bars represent the standard deviations of at least three biological repeats. **(D)** Endogenous expression of selected genes in HEK 293T cells transiently over-expressing c-Myc. Results are normalized to cells transfected with an empty vector (MIG-W) and on reference genes and are presented as averages and standard deviations of independent biological replicates. ^*^ = *p* < 0.01.

To determine c-Myc DNA binding activity gel shift assays were carried out. Note, the yield of nuclear extracts from lung tissue was too small to perform EMSA assays while c-Myc transgenic liver cancer is an abundant source of nuclear proteins as previously reported [8]. In addition, nuclear extracts of HeLa cells served as a positive control. A total of 15 c-Myc binding sites were investigated and EMSA assays confirmed binding activity at gene specific promoters, albeit at different level when individual promoter sequences were studied as exemplified for the Gjb3 (high) and Dsg2 (low) EMSA probes (Figure 2A).

### ChIP assays to determine c-Myc DNA binding activity at gene specific promoters

Moreover, in order to analyze c-Myc DNA binding activity at a cellular level, ChIP assays in human HEK 293T cells were performed upon transient transfection with an empty vector (MIG-W) or a c-MYC over-expressing plasmid (MIG-MYC). A remarkable enrichment of c-MYC-bound signals in the promoter of all selected targets (ADAM19, MAP2K1 and TRAF4) was observed upon over-expression of c-MYC (Figure 2B).

### Chromatin-IP sequencing of human cell lines

We interrogated the publically available ENCODE datasets to determine recruitment of c-Myc at promoters of genes found to be regulated in transgenic lung tumors. c-Myc ChIP-seq data deposited in the UCSC Genome Browser (ENCODE) were retrieved, and data of 7 human cell lines (based on hg19 database) and 2 murine cell lines (mm9 database) derived from 10 different experiments (8 from human and 2 from murine cells) were analyzed for c-Myc binding sites. The findings were compared with orthologue gene promoters and other genomic sequences of differentially expressed genes determined for lung tumors of c-Myc transgenic mice. As shown in Supplementary Table 3 c-Myc binding was confirmed in 76% (56% in murine cells) and 77% (57% in murine cells) of human-derived up-and down-regulated genes, respectively. Fortuitously, the EMSA data obtained with nuclear extracts of the positive control (HeLa cells) could directly be compared with the ChIP-seq data for HeLa cells deposited in the ENCODE database, and this comparison revealed c-Myc binding to be more frequent in up-regulated genes (we observed ChIP-seq positive signals in 52% and 35% of up-regulated and down-regulated DEGs, respectively). Altogether, the EMSA, ChIP and ChIP-seq data agreed (Figure 2A–2B, Supplementary Table 3), therefore evidencing c-Myc recruitment to novel candidate genes.

### c-Myc-dependent activation of selected target genes

A reporter assay was developed to examine functional importance of the newly identified c-Myc binding sites at gene specific promoters. The assay included the PCR amplification of 2kb fragments of Adam19, Traf4 and Map2k1 murine genes that were cloned into a dual luciferase-based reporter retroviral vector (pCZ-REN-P-LUC) as recently reported [6]. While all three genes were up-regulated in the array experiment, only Traf4 and Map2k1 contained consensus E-boxes elements, whereas Adam19 presented E-box-like motives. Consistent with this fact Traf4 and Map2k1 exhibited stronger c-Myc binding according to the ChIP-seq data (summarized in Supplementary Table 3). A further objective of the gene reporter assay was to determine whether c-Myc over-expression would result in promoter and transcriptional activation of the target gene. Vice versa we wished to explore indirect effects of c-Myc in the transcriptional control of these genes. To this effect, transient transfection in HEK 293T cells included a c-Myc expression vector and as shown in Figure 2C evidence was obtained for Traf4 and Map2k1 constructs to be strongly or moderately activated by c-Myc, while Adam19 was not responsive. The results suggest that Traf4 and Map2k1 can be considered as direct gene targets for c-Myc, while the observed up-regulation in tumors for Adam19 appear to be c-Myc-independent or possibly related to additional regulatory elements outside of the 2kb region that was cloned for the reporter assays. The findings also suggest that genes lacking consensus regulatory sequences are not responsive even with ectopically expressed c-Myc protein.

Expression of the c-*Myc* target genes *Adam19, Map2k1, Traf4* and *Fas* upon c-Myc over-expression was studied (Figure 2D). HEK 293T cells were transiently transfected with an empty vector (MIG-W) or a c-Myc over-expressing plasmid (MIG-MYC). The results show that in response to c-Myc over-expression only human *Traf4* is transcriptionally activated, while *Adam19* and *Map2k1* were not affected (Figure 2D). Included in this analysis is *Fas* that was down-regulated in transgenic lung tumors; note, ectopic expression of c-Myc directly repressed its expression. ChIP-seq data from human and murine cell lines also revealed c-Myc binding to the *Fas* gene.

### Correlation between gene expression, *in silico* mapped E-box motives, EMSA band shift assays, gene reporter, ChIP-seq and ChIP qPCR data

Table 2 informs on the different sets of results obtained from various experiments and methodologies. The presence of E-box or E-box-like motives (indicated in the second and third column) in the regulatory regions of genes that were found differentially expressed in PLACs in response to c-Myc over-expression was correlated to experimental observations: a) c-Myc DNA binding in cell-free extracts with radioactive-labeled probes containing the E-box or E-box-like motifs found in the promoter of regulated murine genes (EMSA, forth column), b) c-Myc-dependent transactivation via Luciferase assays on reporter vectors containing 2kb of the regulatory regions of the murine genes (fifth column), c) c-Myc DNA binding retrieved from ENCODE database (ChIP-seq, sixth column) and d) validated direct c-Myc DNA binding in a human cellular system via ChIP qPCR (last column). In all, 76 E-box or E-box-like containing regulatory sequences were experimentally tested. This includes data from our recent publications [6, 7]. A good correlation between the presence of a consensus E-box element (CACGTG) and strong binding in EMSA assays (36/40 vs 1/36) was observed (see also Table 2A). Moreover, 10 regulatory regions (2kb each, comprising 1kb upstream and 1kb downstream of TSS) of c-Myc inducible genes were examined in gene reporter assays. For 9 of them, E-box or E-box-like sequences were determined and DNA binding through EMSA assay was compared with c-Myc-induced transactivation in gene reporter assays. Taken collectively, the presence of a consensus E-box element was nearly always associated with c-Myc responsiveness (7 out of 8 cases, only Prc1 regulatory region despite showing an E-box motif was not activated by c-Myc over-expression) (Table 2B). Among the 2 remaining targets that do not contain a consensus E-box, one was c-Myc responsive (Birc5), indicating that other *cis*-element features (sequence context) or transcription factors/co-factors might participate in c-Myc responsiveness. Lastly, published ChIP-seq data of 7 different human cell lines from 8 independent experiments and ChIP qPCR from c-Myc over-expressing HEK293T cells were analyzed. There was clear evidence for the tested genes to be bona fide targets of c-Myc and given that ChIP-seq revealed similar c-Myc binding activity in different human cell lines, highlighting that an evolutionary conservation of the c-Myc transcriptional network in orthologue promoters of human and mouse genes can be proposed.

**Table 2:** Correlation between presence of the E-box consensus sequences (CACGTG) and c-Mycdependent transcriptional responses

A. Number of E-box (yes) or E-box like (no) motives tested by EMSA		
E-box consensus (^#^tested)	EMSA score^1^	Gene specific sequences
no (36)	-	17
	+	16
	++	2
	+++	1
yes (40)	-	0
	+	0
	++	4
	+++	36

B. Number of E-box or E-box like motives, strength of DNA binding in EMSA assays, c-Myc-dependent transactivation, ChIP-seq data from the literature and ChIP qPCR in human cells over-expressing c-Myc						
Gene	^#^ E-box or E-box-like1	E-box Consensus	EMSA score^2^	Luciferase Assay^3^	ChIP-Seq^4^	ChIP qPCR^5^
Adam19	3 (0)	no	+	1.12 ± 0.07	no	yes
		no	n.a.			
		no	n.a.			
Map2k1	5 (2)	yes	n.a.	1.50 ± 0.07	yes	yes
		no	n.a.			
		yes	n.a.			
		no	+/++			
		no	n.a.			
Prc1	3 (1)	no	n.a.	0.99 ± 0.01	yes	yes
		yes	n.a.			
		no	+			
Traf4	3 (1)	no	+	3.17 ± 0.60	yes	yes
		no	n.a.			
		yes	n.a.			
Hk1	4 (4)	yes	n.a.	3.0 ± 0.58	yes	yes
		yes	+++			
		yes	n.a.			
		yes	n.a.			
	
Rpsa	3 (2)	no	n.a.	1.73 ± 0.05	yes	yes
		yes	n.a.			
		yes	+++			
Npm3	3 (2)	yes	n.a.	1.60 ± 0.10	yes	yes
		no	n.a.			
		yes	+++			
Rcl1	3 (1)	yes	n.a.	1.71 ± 0.05	yes	yes
		no	n.a.			
		no	+			
Srm	6 (5)	no	n.a.	2.37 + 0.07	yes	yes
		yes	n.a.			
		yes	n.a.			
		yes	n.a.			
		yes	+++			
		yes	n.a.			
Birc5	0	no	n.a.	1.30 + 0.06	yes	yes

^1^Number of E-box, or E-box-like, sequences (indicated in brackets is the number of canonical E-boxes, CACGTG) within 2kb (centered around the TSS) of the regulatory regions found in genes differentially expressed in PLACs. They are fully listed in the adjacent column from the most distal to the more proximal site in respect to the TSS.

^2^Value of the signal obtained with EMSA assays. “-“ = lack of shifted bands; “+” = weak, “++”medium, “+++” strong bandshift signal. “n.a.” stands for “not analyzed” sequence.

^3^Average fold changes and standard deviations of the reporter (containing the putative c-Myc responsive region) obtained in human cells with ectopic over-expression of c-Myc as compared to control cells with endogenous c-Myc expression.

^4^ChIP-seq data were retrieved by analyzing eight independent experiments with human cell lines deposited in the ENCODE database from UCSC genome browser. “Yes” in the table indicates overlapping c-MYC binding sites that were identified in at least three independent experiments.

^5^ChIP qPCR validated c-Myc occupancy within the promoter region of candidate genes in cells ectopically over-expressing c-Myc.

Overall, the RT-PCR, Western blotting, EMSA, ChIP and gene reporter assays are consistent.

### Kaplan–Meier survival statistics

**Figure 3:**
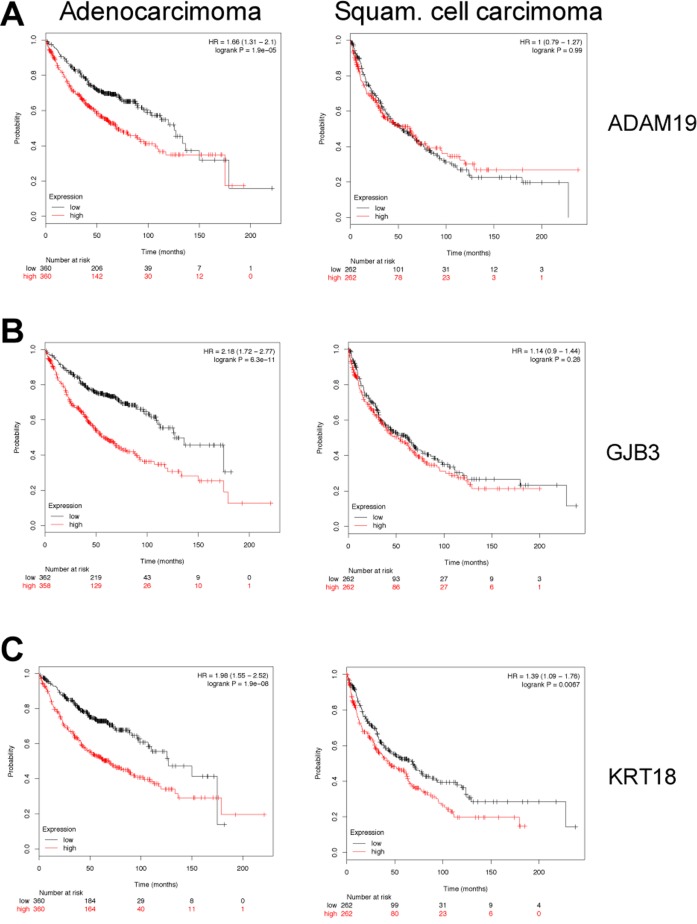

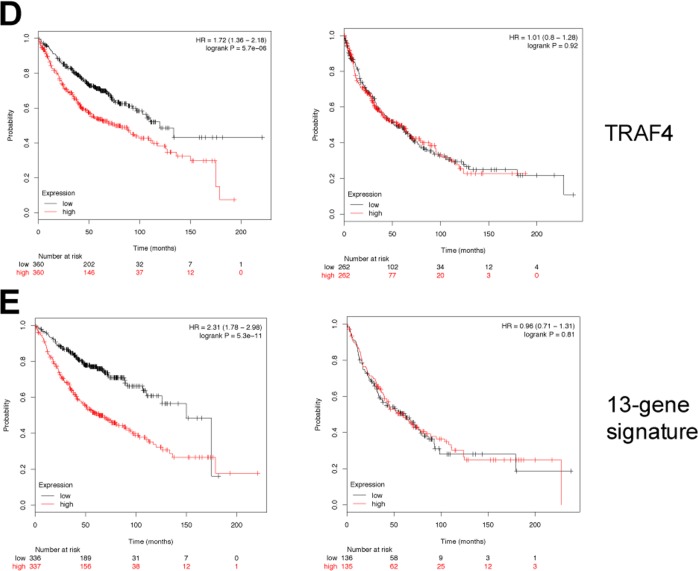
Kaplan–Meier survival statistics of c-Myc induced target genes (**A–E**) Kaplan–Meier curves of lung adenocarcinoma (left panels) and lung squamous cell carcinoma (right panels) were generated using KM plotter tool by considering the expression of the up-regulated genes in c-Myc transgenic lung tumors. Red and black curves always represent patients with high and low expression levels, respectively, of the selected gene. Given underneath each curve is the number of patients considered. Additionally, the Hazard Ratios and the *p*-values are shown in the plot. (E) A “16-gene signature” of the up-regulated genes was evaluated for clinical relevance in human lung cancers (see Supplementary Table 3).

**Figure 4:**
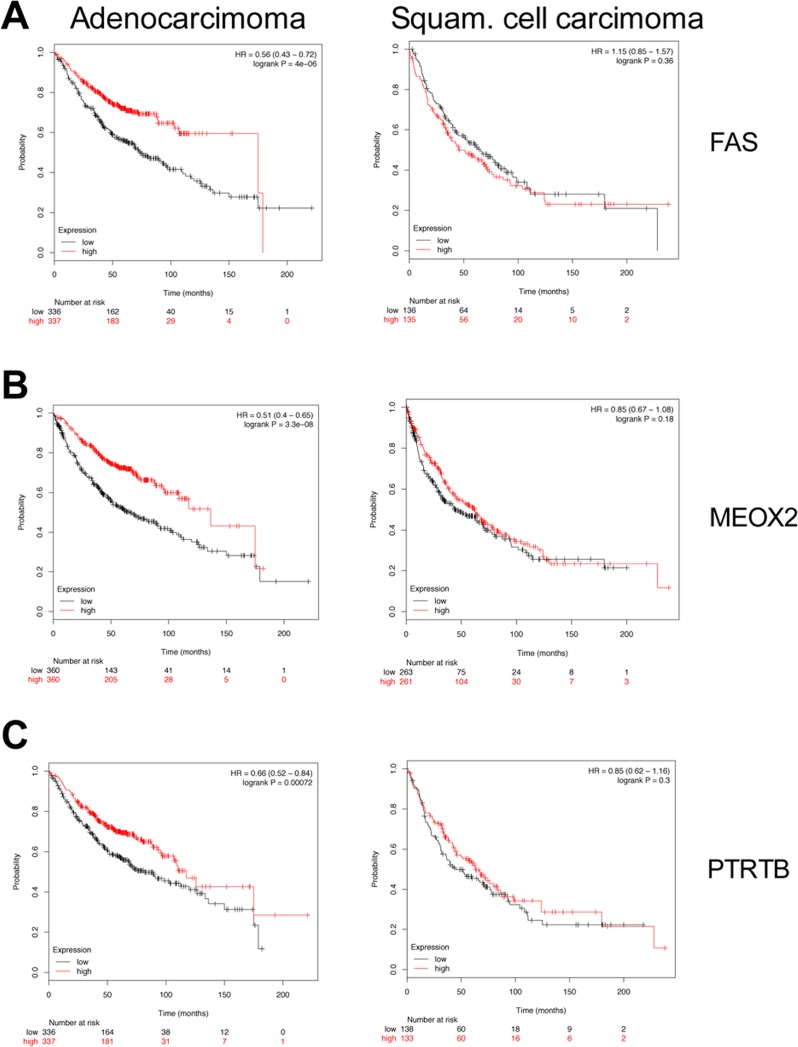

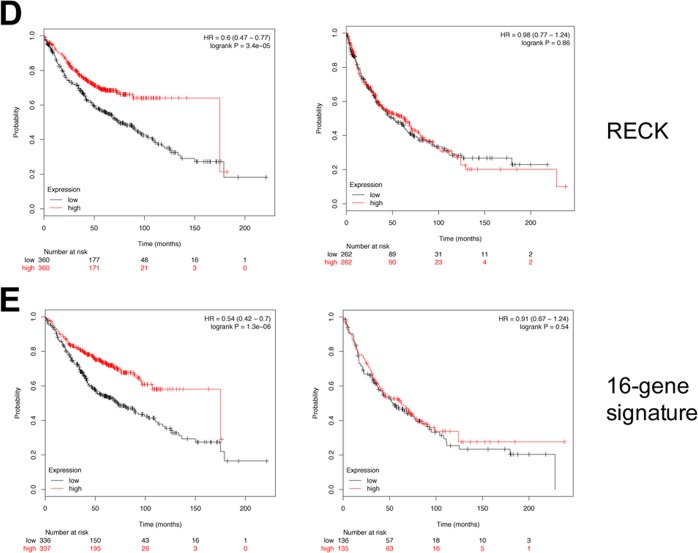
Kaplan–Meier survival statistics of c-Myc repressed target genes (**A–E**) Kaplan–Meier curves of lung adenocarcinoma (left panels) and lung squamous cell carcinoma (right panels) were generated using KM-plotter tool by considering the expression of down-regulated genes in c-Myc transgenic lung tumors. Red and black curves always represent patients with high and low expression levels, respectively, of the selected gene. Given underneath each curve is the number of patients. Additionally, the Hazard Ratios and the *p*-values are shown in the plot. (E) A “13-gene signature” of strongly down-regulated genes (FC < –10 as average of the c-Myc transgenic PLAC tumors) was evaluated for clinical relevance in human lung cancers (see Supplementary Table 3).

To determine clinical significance of murine c-Myc-regulated lung tumor genes Kaplan–Meier survival statistics were calculated for a large cohort of lung cancer patients. Given that the microarray platform used in the present study is identical to the one stored within the KM-plotter database, direct comparisons could be made. We generated Kaplan–Meier curves for 720 lung adenocarcinoma and 524 squamous cell carcinoma patients and determined Hazard Ratios (HR) and *p*-values for statistically significant associations related to 14 up- and 46 down-regulated genes. The data are summarized in Supplementary Table 4, and depicted in Figure 3 are survival curves for a selection of up-regulated genes (KRT18, TRAF4, ADAM19 and GJB3). Importantly, while high expression of the genes correlated with poor prognosis (Figure 3A–3D, left panels), no correlation was obtained when patients diagnosed with squamous cell carcinoma were considered (Figure 3A–3D, right panels) thus demonstrating specificity for adenocarcinomas. Moreover, a combined 13-gene signature of up-regulated genes (highlighted in green in Supplementary Table 4) showed a statistical significant worse outcome when lung adenocarcinoma patients expressed higher levels of the selected genes (Figure 3E, right panel). It should be noted that a higher expression of GJB3 yielded a similar HR as compared to the 13-gene signature (HR 2.18 vs 2.31) and the mean survival was also similar (about 50 months). Subsequently, down-regulated genes (FAS, PTPRB, MEOX2 and RECK) were considered where lower expression of the genes correlated with poor prognosis in lung adeno- but not so in squamous cell carcinoma patients (Figure 4A–4D, left panels). Evidently, the murine transgenic lung cancer model recapitulates c-Myc regulation in human lung adenocarcinomas, and the observations are consistent with c-My-dependent gene regulations in transgenic tumors where high expression is associated with poorer prognosis, while the opposite is seen for down-regulated genes.

We next defined a gene-signature for strongly down-regulated genes (FC ≤ 10-fold) in lung cancer tissues in mice (highlighted in green in Supplementary Table 4). Remarkably lower expression of the 16-gene signature was associated with poorer prognosis in lung adenocarcinoma patients (Figure 4E, left panel).

Lastly, since Miz1 can cooperate with c-Myc in the transcriptional regulation of targets in cancer tissues, the link between expression of this gene in lung cancer samples and survival was also tested. Interestingly, lung adenocarcinoma patients with higher expression of Miz1 showed a poorer prognosis, while no effects were seen with squamous cell carcinoma patients (Supplementary Figure 1, respectively left and right panels)

### RT-qPCR validation of candidate genes in human lung cancer and matched normal tissue

**Figure 5:**
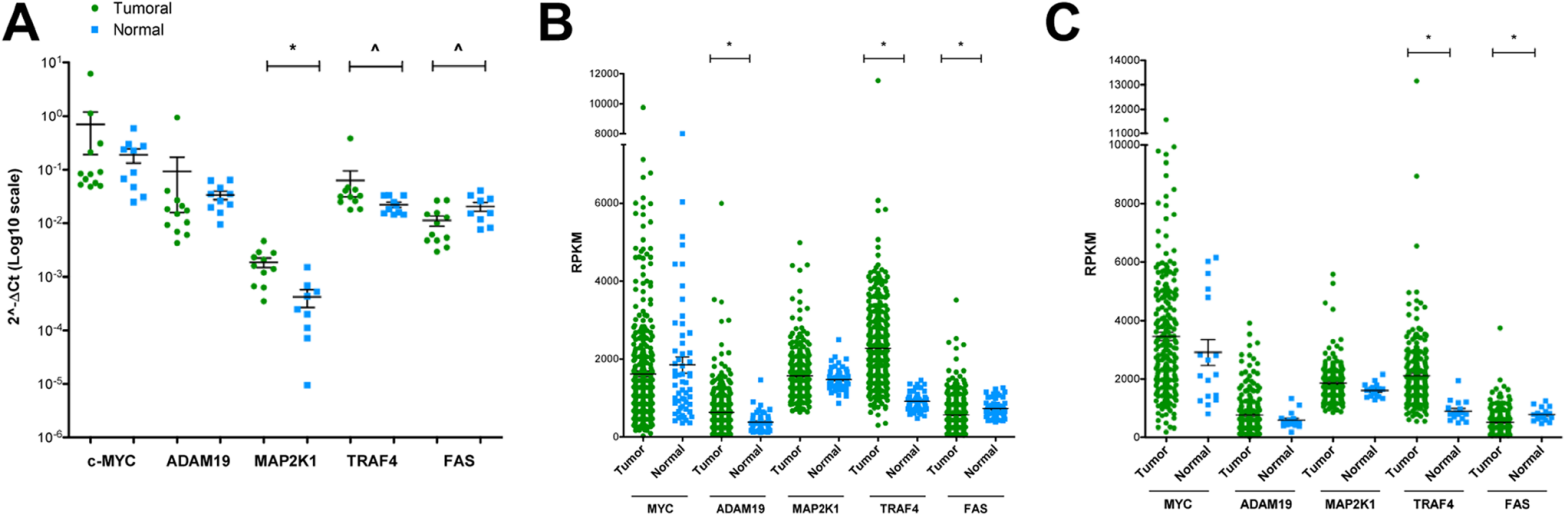
RT-qPCR validation of candidate genes in human lung cancer and matched normal tissue (**A**) RT-qPCR analyses for the expression of selected genes in 12 lung cancer patients and matched normal lung tissue of the same resection material were performed. Results are presented as scatter plots with the relative expression of each gene of interest normalized on reference genes (YWHAZ and ACTB). (**B–C**) Relative expression values from RNA-seq data (TCGA) of selected genes in 355 lung adenocarcinoma (green circles, compared to 57 normal samples, light blue squares) (panel B) or 220 lung squamous cell carcinoma (green circles, compared to 17 normal samples, light blue squares) (panel C). Shown are the means and standard errors. ^ = *p* < 0.05; ^*^ = *p* < 0.01.

Based on the histological phenotype a total of 12 lung cancer patients were selected and for 10 patients matched normal lung tissue was studied as well. Transcript expression of ADAM19, MAP2K1, TRAF4 and FAS along with c-MYC was evaluated by RT-qPCR. Consistently, higher expression in tumor samples for two of the up-regulated genes (MAP2K1 and TRAF4) and lower expression in tumor samples for down-regulated genes (FAS) was observed to evidence their tumor specific regulation (Figure 5A).

Encouraged by these observations, a larger cohort of lung cancer patients originally investigated by the TCGA Consortium was retrieved from the publically available repository. We considered 355 lung adenocarcinoma patients and compared the data to 57 healthy controls. Alike we considered 220 cases of squamous cell carcinoma patients and compared the data to 17 healthy controls. Once again we confirmed tumor specific regulations for up- and down-regulated genes (Figure 5B–5C). In lung adenocarcinomas, however, MAP2K1 did not reach statistical significance (Figure 5B), while in squamous cell carcinomas a similar pattern can be seen for MAP2K1 and ADAM19 (Figure 5C). Intrigued by these observations, we extended the analysis to HGFAC and SPINT1; note, these were up-regulated in murine lung cancer tissues and are potential c-Myc target genes. When compared to normal lung tissues both genes were expressed at significantly higher levels in tumors of lung adenocarcinoma patients; however, SPINT1 was also up-regulated in squamous cell carcinoma (Supplementary Figure 2, (panels A-B) lung adenocarcinoma; (panels C-D) lung squamous cell carcinoma).

### Oncogenomics of c-Myc transgenic tumors reveal bona fide drug targets

The genomic study revealed 24 up-regulated genes in c-Myc transgenic tumors (Table 1) and their regulation was considered in lung cancer patients. The plotted Kaplan–Meier curves revealed 12 of them to be associated with poor outcome when over-expressed in lung tumors. The expression of the coded proteins was also examined by IHC. For this purpose data was retrieved from the publically available Human Protein Atlas repository (HPA, Supplementary Table 4). The data agreed even though the number of patients with high expression of the protein varied for the different targets. For instance, c-Myc was induced nearly 60-fold in large transgenic tumors and 9 out of 12 patients examined also had high expression of the protein (Supplementary Table 4). Alike, expression of the serine protease inhibitor Kunitz type 1 was consistently up-regulated in c-Myc transgenic tumors and the protein was highly expressed in 11 out of 12 patients examined. Importantly, independent research discovered an interaction between Spint1 and membrane-bound serine proteinases to support EMT transitions [9], and inhibition of Spint1 was shown to be of therapeutic benefit by increasing tumor specific immune responses [10]. A further example is thimet oligopeptidase 1 (THOP1) and the protein was highly expressed in 8 out of 9 patients examined. Note, low THOP1 expression predicts poor prognosis of metastatic NSCLC [11]. Moreover, in large c-Myc transgenic tumors Ros1 was highly induced and the FDA recently approved Crizotinib for the treatment of ROS1-positive tumors in NSCLC patients [12]. Conversely, up-regulation of keratin complex 1 functions as a tumor suppressor that is targeted by the early growth response 1 transcription factor [13] and the protein was highly expressed in 10 out of 12 patients examined. A similar result was obtained when desmocollin was considered. The protein functions as a tumor suppressor [14] and was highly expressed in 10 out of 12 patients examined. A similar conclusion can be drawn when considering the other desmosomal adhesion molecule, i.e. desmoglein 2 which was induced as well. Loss of the protein promotes tumorigenesis [15] and high expression of the protein was observed in 9 out of 10 patients examined.

Further examples include the adenosine receptor Adora2b that was shown to play a major role in tumor progression by suppressing tumor specific immune responses [16] as well as connexin 31 also known as gap junction membrane channel protein β3. In a recent review dysregulation of connexins in cancer have been highlighted and are currently validated for druggability [17]. Finally, Batimastat and Marimastat, i.e. Adam19 inhibitors, were extensively evaluated in clinical trials as anti-neoplastic agents; however, difficulties in drug administration (Batimastat) and insufficient therapeutic efficacy (Marimastat) halted their development.

## DISCUSSION

c-Myc is an extremely versatile transcription factor that binds literally to thousands of genomic loci [18–20]. Given that c-Myc is deregulated in many cancers an identification of c-Myc target genes and their downstream effectors will be of critical importance for an understanding of its transforming abilities. We recently reported the decoding of c-Myc networks of cell cycle and apoptosis regulated genes in a transgenic mouse model of papillary lung adenocarcinomas, and our continues efforts identified c-Myc targeted regulators of cell metabolism to provide opportunities for targeted therapies [6, 7]. We now conclude our oncogenomic study by focusing on c-Myc's ability to influence extracellular signaling, adhesion, angiogenesis and invasion. Based on a range of experimental studies we validated c-Myc targets and established its clinical relevance by considering Kaplan–Meier survival statistics in a large cohort of cancer patients. Finally, several of the novel c-Myc targets are interesting candidates for the development of molecularly targeted therapies, and this include inhibition of the highly up-regulated genes in lung cancer tissues such as Spint1, Ros1, Adora2b, Gjb3, amphiregulin and Adam19 which were also highly expressed in human tumor tissues as protein.

### Incapacitating extrinsic pathways of growth-inhibitory signals

With c-Myc transgenic lung tumors gene expression changes were specifically associated with repressed death receptor signaling and the up-regulation of uniquely expressed transcripts such as Traf 4 known to inhibit Fas-induced apoptosis [21]. The repressed expression of the pro-apoptotic transcription factor Hoxa5 in lung tumors is another important finding. This protein sensitizes cells more than 100-fold to TNF-alpha-induced cell death that is mediated by caspases 2 and 8 [22]. Its re-expression causes apoptotic cell death in breast tumor cells and Hoxa5 is typically silenced in breast cancer [23]. The promoters of the newly identified candidate genes Tnfrsf6, Traf4 and Hoxa5 carry c-Myc binding sites and regulation of the genes by c-Myc confers resistance to apoptosis induced by cytokines like FasL and TNFalpha.

### Evading the TGF-beta growth-suppression signaling

Several components of the TGF-beta (TGFβ) signaling pathway were repressed which play a role in the control of cell proliferation by either blocking cell cycle progression through G1 or by stimulating apoptosis. Down-regulated genes in lung tumors included the type I receptor for TGFβ ligand Acvrl1 and the TGFβ co-receptor endoglin as well as TGFβ1 and Bmp6. The presence of c-Myc recognition sites in the transcription start-site of Acvrl1 makes this gene a likely c-Myc target. Furthermore, expression of Bmp6 was lost in all lung tumors of transgenic mice as was expression of dermatopontin. The latter codes for a component of the extracellular matrix and increases the cellular response to TGFβ [24]. Besides, repression of the transcription factor Zfhx1a, an important regulator of TGFβ/BMP signaling that synergizes with SMAD to induce growth arrest, was observed [25]. Furthermore, the TGFβ inhibitory growth factor Gadd45b was repressed, which functions as a positive effector of TGFβ-induced apoptosis [26]. Strong repression of Gadd45 was also observed in RAT-1 c-Myc over-expressing cells [27], and repression of these genes disrupts the growth-inhibitory signaling of TGFβ proteins in lung tumors.

### Cell survival signaling by IGFR

Apoptosis can be blocked by exogenous survival factors such as insulin-like growth factor-1, which binds to cell-surface receptors to activate the PI3 kinase-Akt/PKB signaling pathway. In c-Myc transgenic lung tumors survival signaling was sustained through repression of Igf-binding proteins 4, 5 and 6. The proteins sequester IGF and regulate their biological availability but may also exert IGF-independent growth-inhibitory effects. The observed repression of IGFBPs increases the availability of IGF and therefore contributes to an anti-apoptotic signaling via IGF receptors. This notion is also supported by the fact that among different growth factors tested only IGF proved to be a survival factors for c-Myc overexpressing fibroblasts [28]. Furthermore, down-regulation of repressors of the IGF1R signaling was observed most notable Socs2, which interacts with IGF1R to suppress IGF1-induced growth [29] and NFκB-dependent signaling. IGFR-mediated survival signaling is sustained by inhibiting negative regulators of this pathway.

### Activation of tyrosine kinase receptors

The epidermal growth factor receptor plays a key role in transmitting signals of cellular growth and proliferation. In transgenic lung tumors amphiregulin was strongly up-regulated, and this autocrine growth factor binds to the Egfr to stimulate proliferation and survival signaling in human lung epithelial cells [30]. Notwithstanding, amphiregulin inhibits apoptosis in NSCLC through activation of an IGF1R-dependent pathway [31]. Furthermore, amphiregulin facilitates G-protein-coupled receptor (GPCR)-mediated transactivation of EGFR signaling and was shown to induce cellular proliferation and motility in squamous cell carcinoma [32]. A regulatory loop exists whereby secretion of amphiregulin can be induced by an activated EGFR in bronchiolar epithelial cells [33] and through IGF1R signaling in NSCLC [31]. Given its unique role in lung morphogenesis [34], amphiregulin plays a decisive role in lung cancer by stimulating autocrine growth and the suppression of apoptosis.

A further up-regulated component of the EGFR/Ras/Raf/Mek/Erk signaling cascade was Map2k1 (Mek), while Rassf5, the pro-apoptotic Ras effector, was repressed to attenuate Ras-mediated apoptotic signaling. In fact, in 80% of human lung adenocarcinomas Rassf5 is not expressed and was shown to be specifically silenced in lung tumor cells [35]. Additionally, transcripts coding for phosphatases such as Dusp1, Ptpre, Ptpns1, Ptprb and Ptprg were repressed. The de-phosphorylation of signaling proteins like MAPKs will attenuate receptor tyrosine kinase-coupled signaling [36, 37].

Among uniquely expressed genes in c-Myc-induced tumors was the proto-oncogene Ros1 that codes for a trans-membrane tyrosine kinase and may function as a growth factor receptor. Little is known about Ros1 biology in healthy tissue and Ros1 ko mice not developing a specific phenotype [38]. Although the Ros1 protein is not expressed in normal human lung tissue, ROS1 gene fusions with oncogenic activity are reported to occur in about 1–2% of NSCLC patients and are amenable to the development of molecular targeted therapies [39].

Another gene uniquely expressed in tumors is HGF activator, a secreted protease that converts precursor of HGF to its active form and functions as a potent mitogen for epithelial cells to induce angiogenesis via c-Met signaling. Up-regulation of HGF activator in human lung adenocarcinomas was reported [40], and there is strong evidence for the MET/HGF axis to play a major role in the development of primary and acquired chemoresistance [41]

The unique expression of these genes in tumors is of therapeutic utility and the development of inhibitors of Ros1 fusion proteins and of Hgf-dependent and Hgf-independent Met activation is actively pursued. Besides, up-regulation of Adam19 is another important finding. This member of the disintegrin and metalloprotease family is involved in signal processing of trans-membrane growth factor precursors and a key component to foster activation of EGFR signaling [42]. Induced Adam19 expression is frequently seen among different cancer cell lines and is therefore a bona fide target for the treatment of cancer [43].

### Activation of Wnt/beta-catenin signaling

Evidence was obtained for Wnt signaling to be affected by c-Myc. In tumor tissues enhanced expression of Arhu, a member of the Rho family that mediates in part the effects of Wnt1 signaling in the regulation of cell morphology, cytoskeleton organization and cell proliferation [44] was observed. Moreover, unique expression of Frat2, a positive regulator of Wnt signaling and stabilizer of beta-catenin [45] was observed while expression of the transcription factors Tcf7 and Sox7 was repressed, which are negative regulators of Wnt/beta-catenin-induced gene transcription. Down-regulation of these genes was associated with malignant transformation. Overall, modulation of Wnt signaling contributed to promotion of cell proliferation, cytoskeleton dynamics and motility of tumor cells.

### Regulation of cytoskeleton, motility and invasiveness

The ability of malignant cells to migrate and invade surrounding tissues is associated with changed motility based on modifications of the cytoskeleton. Here the Rho family of proteins plays an important role and several genes involved in cytoskeleton dynamics and transformation were changed in expression and included the strongly up-regulated proto-oncogene Ect2. This growth-regulatory molecule is able to regulate cell cycle as a guanine nucleotide exchange factor and also activates the Rho family of Ras-related GTPases thereby remodeling the actin cytoskeleton in support of cellular transformation [46].

In c-Myc transgenic lung tumors expression of the tumor suppressor Vsnl1, i.e. a member of the neuronal Ca^2+^ sensor protein family was highly repressed (Supplementary Table 1). Vsnl1 decreases RhoA and MMP-9 activity through cAMP-mediated signaling [47] and therefore may enhance the malignant phenotype. Down-regulation of calpain-2, an intracellular protease required for proteolysis of the cytoskeleton and focal adhesion proteins, was shown to restrict membrane protrusions and lamellipodia dynamics [48] to further strengthen pro-migratory capacity of lung tumor cells.

### Cell junctions and adhesion

Cell-cell and cell-extracellular matrix (ECM) junctions do not only connect cells mechanically through adhesion, but also transmit signals to the cell interior, thereby playing an important role in the regulation of cell survival, proliferation and migration.

In transgenic lung tumors induced expression of mucin1 was observed and this transmembrane glycoprotein functions as a tumor antigen [49] and is part of the Wnt/beta-catenin signaling [50]. Over-expression of mucin1 is associated with invasiveness in breast [51] and colon cancer [52].

Furthermore, up-regulation of the intercellular adhesion molecule 1, which is the only known direct ligand of the mucin extracellular domain, is another important finding. It was shown earlier that mucin1 interacts with Icam1 and produces calcium-based signals to affect cytoskeletal dynamics and increased cell motility [53]. Conversely, the strong repression of the junction proteins, cadherin-5, protocadherins alpha 4 and 6 may contribute to cell proliferation and migration in lung tumors. Additionally, regulation of several genes implicated in cell-ECM interactions was observed and included repressed fibulin-1, an extracellular matrix (ECM) protein affecting Erk-mediated signaling cascades thereby inhibiting motility and invasiveness of cancer cells [54] and Lama3&4, e.g. ECM glycoprotein also shown to be repressed in various human cancers [55]. The decreased expression of matrix receptor alpha8 integrin, which negatively affects cell migration and proliferation, is another example of altered cell-ECM interactions in lung cancer.

### Degradation of extracellular matrix

Cell migration necessitates degradation of extracellular matrix. In c-Myc transgenic lung tumors the gelatinase-associated protein lipocalin 2 was induced up to 7-fold. This regulator of matrix metalloproteinase is involved in invasion of tumor cells [50, 56] and inhibition of the protein impairs breast tumorigenesis and metastasis [57]. Conversely, Reck was highly significantly repressed (up to 6-fold) and the protein functions in the suppression of invasiveness of cancer cells through inhibition of MMP release [56]. Likewise, up-regulation of the metalloproteinases Adam19 and Thop1 are notable findings. Thop1 is a metallo-endopeptidase and its higher expression in NSCLC patients is associated with better survival [11]. Additionally, the serine and cell surface protease hepsin was about 5-fold up-regulated among small, middle sized and large tumors. This protein causes disorganization of basement membrane to promote progression of metastasis in a mouse prostate cancer model [58] whereas targeted inhibition of hepsin blocks prostate cancer bone metastasis [59]. Lastly, gene transcription of the extracellular enzyme lysyl oxidase (Lox) was repressed to suggest inhibition of cross-linking of collagen and elastin fibers in ECM that makes the matrix fragile and prone to tear. Although LOX is over-expressed in several cancers and was shown to enhance tumor progression, its reduced expression in some cancers indicates a tumor-suppressor function [60, 61].

### Gap junctions

Over-expression of connexins 43 and 31 in lung tumors revealed an unsuspected role of c-Myc in cell-cell communication. These connexins were found to contain c-Myc binding sites adjacent to the transcription start site and function as intercellular channels to allow the exchange of small molecules (such as inorganic ions, sugars, nucleotides, intracellular mediators like cyclic AMP and IP3) among coupled cells. Connexin 43 is essential for survival, and its expression was altered in malignant tissues. An earlier report suggests connexins can be induced through Ras signaling [62] and the consequences of perturbed connexin 31 expression on cell proliferation and cell death [63] were already discussed.

### Tight junctions

Tight junctions seal epithelial cells together and function as selective permeability barriers. They are formed exclusively by integral membrane proteins of the claudin family and their role in cancer is the subject of ongoing research. A high level of claudin-5 gene expression was observed in healthy control animals but declined progressively and eventually was absent in large tumors. This suggests loosening of intercellular adhesion and increased motility. Conversely, gene expression of claudin 2, absent in normal lungs, was strongly up-regulated in tumors, probably by activation of Wnt signaling [64]. Claudin 2 as well as other claudins are involved in the activation of pro-MMP through recruitment of enzymes on the cell surface [65] thereby contributing to invasion.

### Pro-angiogenic signaling

A remarkable nearly 38-fold up-regulation of kininogen in mid-sized tumors was observed (Table 1). This protein functions as a hemostatic factor and exerts its angiogenic effect through signaling of its cleaved product form bradykinin via the B (2) receptor [66]. In small tumors expression of the G-protein-coupled receptor Adora2b was about 6-fold induced to stimulate angiogenesis. Note, a functional cooperation between adenosine A2B and A3 receptors was reported to promote angiogenesis [67]. Furthermore, in all lung tumors the expression of Sema3f was lost. This signaling molecule inhibits Vegf due competitive binding to neuropilin1/2 receptors. Indeed, Sema3f inhibits angiogenesis [68], metastasis and motility of primary tumor cells, and decreased expression of Sema3f is correlated with advanced disease stages and more aggressive tumors in human NSCLC [69], indicating its important role in lung carcinogenesis.

A link of c-Myc to angiogenesis can also be inferred from studies with skin [70], pancreatic β-cells [71] and lymphoma [72] with reversible activation of conditional c-Myc alleles inducing hyper-proliferation, dedifferentiation and angiogenesis.

### Signaling through scaffold proteins

As described above c-Myc activity is linked to several intra- and extracellular signaling pathways. As scaffolding proteins are involved in the spatiotemporal regulation of signaling pathways the present study evidences caveolin-1 and Akap12 (gravin) to be regulated in lung cancer. Their expression is frequently silenced in carcinomas [73] including lung cancer [74]. Importantly, caveolin-1 is the principal structural component of caveolae that harbors growth factor receptors, G-proteins as well as calcium channels and pumps and is thought to be a major regulator of signaling complexes. Loss of caveolin-1 was demonstrated to confer a significant growth advantage to mammary epithelial cells *in vitro* and *in vivo* [75]. Decreased expression of caveolin-1 together with over-expression of Fas is suggestive for membrane rafts to be altered in transformed cells, Here, the function of the transformation suppressor Akap12 seems to be dependent on the ability to reorganize actin-based cytoskeleton architecture to control G1-phase signaling proteins [76]. Scaffold proteins thus guide the cross-talk between mitogenic signals across different signaling pathways and the control of cell cycle. The loss of such spatiotemporal regulators of signaling processes in response to c-Myc may amplify proliferative signals.

Lastly, STRING protein-protein interaction network analysis revealed 59 out of 97 DEGs to interact with each other thus further highlighting possibilities for the development of molecularly targeted therapies through disintegration of the network (Supplementary Figure 3).

**Figure 6:**
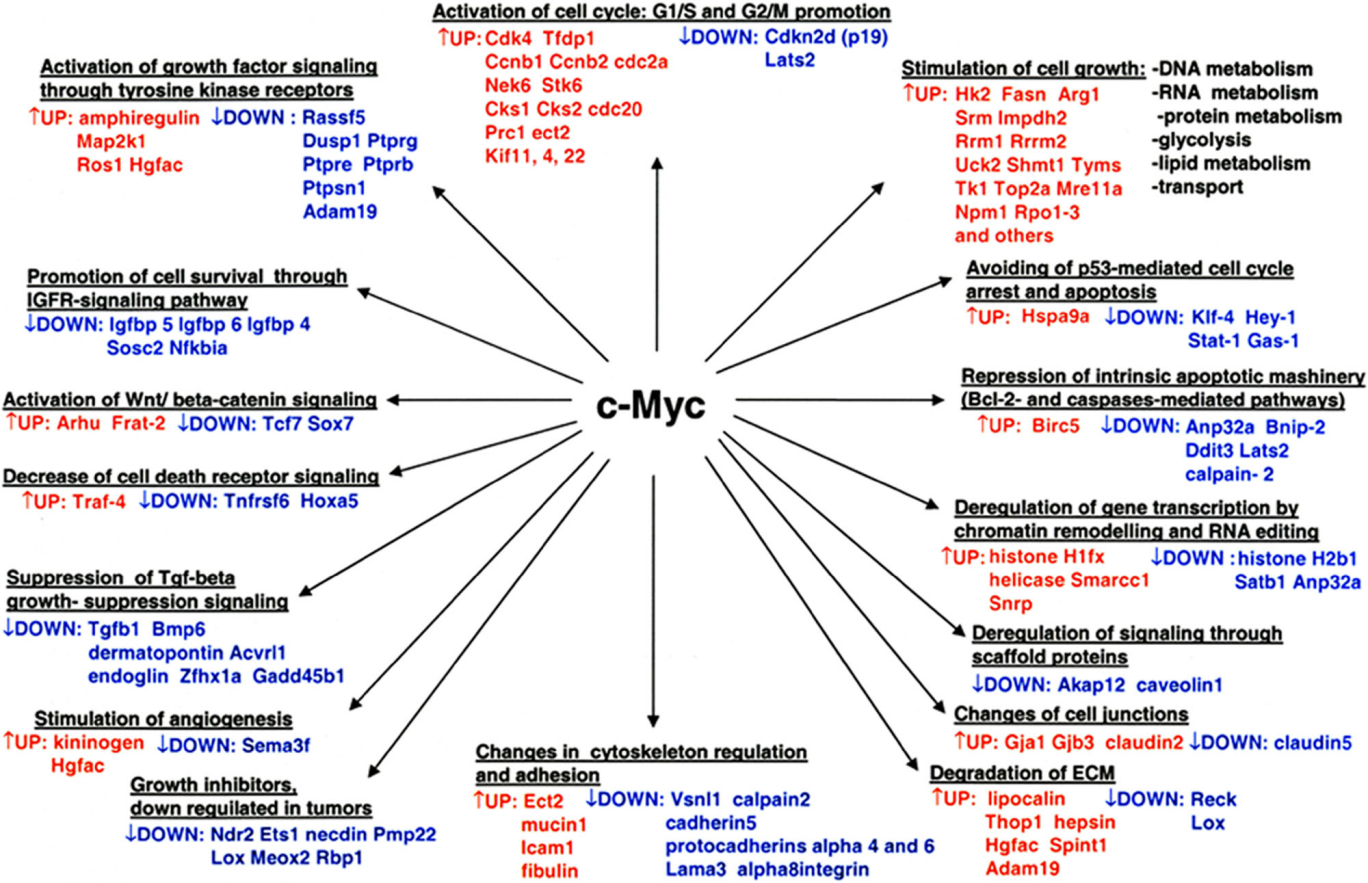
Overview of pathways and novel gene candidates identified in c-Myc-induced lung adenocarcinoma Color coding: red = up-regulated genes; blue = repressed genes.

In conclusion, a transgenic disease model was interrogated that helped to define novel c-Myc targeted regulatory gene networks in papillary lung adenocarcinomas (Figure 6).

Of the many genes regulated 76 E-box or E-box like containing regulatory sequences were experimentally tested at gene specific promoters and a good correlation between the presence of a consensus E-box element (CACGTG) and strong binding in EMSA assays (36/40 vs 1/36) was obtained (Table 2A).

Furthermore, ten regulatory regions (2kb each, comprising 1kb upstream and 1kb downstream of TSS) of c-Myc inducible genes were examined in gene reporter assays. For nine of them, E-box or E-box-like sequences were tested in EMSA assays and DNA binding activity was compared to c-Myc-induced transactivation. The presence of a consensus E-box element was always associated with c-Myc responsiveness (Table 2B). Among the targets that do not contain a consensus E-box motive, two were c-Myc responsive, indicating that other cis-element features (sequence context) or transcription factors/cofactors might participate in c-Myc responsiveness.

Many of the genes identified in c-Myc transgenic lung tumors were similarly regulated in human malignancies thus highlighting the relevance of the disease model in an identification of new c-Myc-responsive and putative c-Myc targets genes. Thus, our study helps to define the c-Myc transcriptome in lung adenocarcinomas. The performed Kaplan–Meier survival statistics demonstrated clinical relevance of the findings to encourage translational research and the development of mechanism-based therapies.

## MATERIALS AND METHODS

### Ethics statement

#### Human lung cancer tissue samples

Research was carried out according to the Declaration of Helsinki as revised in 2013 and ethical approval for the use of liver samples was obtained from the Ethics Committee of Hannover Medical School (Tr/L, 2499). Tumor resection material and matched healthy control tissue from 12 patients (average age 70 years; range 58-84) was considered for RT-qPCR validation of c-Myc candidate genes. Based on the TNM classification the patients were staged as T2B to T3B with lymphnode involvement (ipsilateral, peribronchial and/or ipsilateral hilar and intrapulmonary nodes), and imaging of lung cancer patients revealed atelectasis/obstructive pneumonitis extending to the hilar region. The pattern of tumor growth was histologically defined as 6 cases of acinar, 3 cases of papillary, 3 cases of lepidic growth of which two cases were mixed lepidic/mucoid. All except one case were positive for the thyroid transcription factor 1.

#### Animal studies

All animal work followed strictly the Public Health Service (PHS) Policy on Humane Care and Use of Laboratory Animals of the National Institutes of Health, USA. Formal approval to perform *in vivo* experiments with animals was granted by the animal welfare ethics committee of the State of Lower Saxony, Germany (‘Lower Saxony State Office for Consumer Protection and Food Safety’, LAVES). The approval ID is Az: 33.9-42502-04-06/1204.

#### Maintenance of the transgenic mouse line

The development of the SPC/myc-transgenic disease model was reported previously [4]. Essentially, the plasmid is composed of a gene construct with the Surfactant C promoter selectively targeting c-Myc over-expression to respiratory epithelium. Mice were maintained as hemizygotes in the CD2F1-(DBA/2xBalb/C) background, and the transgene was verified by PCR with DNA extracted from tail biopsies with Platinum PCR SuperMix (Invitrogen, Karlsruhe, Germany) and the following primer pair: 5′-CAGGGCCAAGGGCCCTTGGGGGCTC TCACAG, 3′-GGACAGGGGCGGGGTGG GAAGCA GCTCG. The mice developed the full spectrum of atypical adenomatous hyperplasia (AAH) hallmarked by lepidic growth pattern, carcinoma *in situ*, followed by lung adenocarcinomas and solitary tumors with papillary growth pattern.

#### Sample collection and preparation

Mice aged between 9 – 13 months were anesthetized by an overdose of CO_2_ and the lungs were explanted and rinsed with ice-cold physiological saline. The tumors were inspected macroscopically, separated from the surrounding lung tissue and frozen immediately in liquid nitrogen. Normal lung from *n* = 4 non-transgenic mice served as control.

#### Isolation of RNA, production of cRNA, array hybridization and scanning

Total RNA was extracted using the Qiagen RNA isolation kit according to the manufacturer's instructions (Qiagen, Hilden, Germany) and copy RNA was prepared according to the Affymetrix Gene Chip^â^ Expression Analysis Technical Manual (Santa Clara, CA). Further details are given in our recent report [6]. The microarray study is MIAME compliant, and the raw data have been deposited in GEO database (GSE10954; http://www.ncbi.nlm.nih.gov/geo).

#### Gene expression data analysis

A detailed description of the statistical analysis of the gene expression data is given in our recent manuscripts [6, 7].

### Gene expression studies by RT-PCR and RT-qPCR

#### Mouse lung tumors

Total RNA was extracted as described above. Reverse transcription was carried out using Oligo-dT primers (Invitrogen), RNasin (Promega, Mannheim, Germany) and Omniscript (Qiagen) followed by PCR amplification.

PCR reactions were carried out with Taq Platinum PCR Super-Mix Kit (Invitrogen), and amplification products were separated on 1% agarose gels. Densitometric scans were obtained with the Kodak 1D Image Analysis Software. The gene expression values were normalized to beta-actin expression and used to calculate mean expression values. The mean fold change was computed as a ratio between gene expression values for tumor and control non-transgenic lungs.

#### Cell cultures

HEK 293T cells were obtained from ATCC (American Type Culture Collection, LGC Standards, Molsheim, France) and were cultured in DMEM supplemented with antibiotics, L-glutamine and 10% FBS serum. Cells were seeded in 6-well plates and transfected with MIG-empty or MIC-MYC expression vectors using Lipofectamine LTX (Invitrogen, Life Technologies, Milan, Italy). After 24 hours cells were harvested and total RNA was extracted as described above. cDNA was generated from 2mg of RNA using the RevertAid First Strand cDNA Synthesis kit following manufacturer's recommendations (Thermo Fisher Scientific, Milan, Italy). Twenty-five ng of cDNA were used for RT-qPCR experiments to detect expression levels for c-MYC, ADAM19, MAP2K1, TRAF4 and FAS mRNAs. Primers were designed using Primer-Blast tool and tested for efficiency and specificity. YWHAZ and ACTB were used as reference genes. mRNA expression was measured through Kapa Sybr Fast Master Mix (Kapa Biosystems, Resnova, Ancona, Italy) and the CFX384 Detection System (BioRad, Milan, Italy). Expression values were obtained as ΔΔCt calculations normalized on reference genes as previously described [77, 78].

A list of the primer sequences used both for RT-PCR and RT-qPCR reactions is presented in Supplementary Table 5.

#### RT-qPCR validation of candidate genes in human lung cancer samples

A total of 12 patients were selected based on the histological phenotype (papillary, acinar and lepidic growth pattern) and included matched normal lung tissue of the same patient. Frozen samples were cut in 5-10 mm slices using the Leica CM 1850 UV cryostat, and 30 to 100 mg of tissue was collected. Subsequently, the slices were lysed with TRIzol (Life Technologies) and 1.4 mm ceramic spheres (Lysing Matrix D, MP Biomedicals, DBA, Milan, Italy) using the FastPrep-24 homogenizer (MP Biomedicals) (2 cycles, 30’’ on at room temperature and 5’ off on ice). RNA was extracted with chloroform and isopropanol using standard protocols. RNA integrity was checked by gel electrophoresis. Two mg of purified RNA was converted into cDNA, and 25 ng was used for RT-qPCR experiments as described above; expression calculations were generated as ΔCt values normalized to reference genes *ATCB* and *YWHAZ*. Primers are the same used for the experimental procedures as explained in the previous paragraph.

#### Western blotting

Proteins from lung tumors of SPC/c-Myc-transgenic mice and/or non-transgenic animals were extracted and quantified as previously reported [7]. Seventy-five or 100 μg (50 μg in case of HEK293T over-expressing c-MYC) of total protein extracts were separated on 12.0% SDS-polyacrylamide gel and blotted onto PVDF membranes in 25 mM Tris and 190 mM glycine at 4 °C for 2 h at 350 mA or using the semi-dry iBlot transfer system (InVitrogen, Life Technologies). Specific antibodies were used in dilution given in parenthesis for the detection of anti-Traf4 (1:100), anti-hepsin (1:100), anti-Alk1 (= Acvrl1) (1:100), anti-Igfbp6 (1:100), anti-c-MYC (1:1000) and anti-α-Actinin (1:8000) purchased from Santa Cruz Biotechnology, Inc. (Heidelberg, Germany); anti-claudin2 (1:500) purchased from Zymed Laboratories, Inc. (San Francisco, CA, USA) and anti-Adam19 (1:1000) purchased from Cedarlane Laboratories (Burlington, Ontario, Canada). Antigen-antibody complexes were visualized using the ECL detection system NEN Life Science Products (PerkinElmer Life Science, Rodgau-Juegesheim, Germany) or ECL Select kit (GE-Healthcare, Milan, Italy) as recommended by the manufacturer and recorded with the Kodak IS 440 CF (Kodak, Biostep, Jahnsdorf, Germany) and a ChemiDoc XRS+ (BioRad, Milan, Italy).

#### EMSA assays

Electrophoretic Mobility Shift Assays (EMSA) were carried out as described in [6, 7]. Oligonucleotides were annealed and subsequently labeled using [32P] ATP (Perkin, Elmer, Rodgau-Jügesheim, Germany) and T4 polynucleotide kinase (New England Biolabs, Frankfurt am Main, Germany). End-labeled probes were separated from unincorporated [32P] ATP with a Microspin G-25 Column (GE Healthcare, Freiburg, Germany) and eluted. The sequences of the EMSA probes are given in Supplementary Table 6.

#### Bioinformatic search for c-Myc binding sites in the 5′-UTR region of differently expressed genes in tumors

Different positional weight matrices (PWMs), i.e. M01034, M00322, M00118, M00123 and M00615 were retrieved from the TRANSFAC**^®^** Professional rel. 8.3 database [79] (http://www.biobase.de/) and applied to genomic sequences of regulated genes. For this purpose, the UCSC Mouse Genome Browser was used to extract the putative promoter regions of the corresponding genes (www.genome.ucsc.edu/cgi-bin/hgGateway). The first ATG codon was considered as tentative TSS (transcription start site), and 1000 bp upstream and 1000 bp downstream of TSS were extracted. Moreover, the MATCH^TM^ algorithm was used to calculate scores for the matches by use of the so-called information vector. The core similarity cut-offs for the matrices were set to 0.75, and the matrix similarity cut-offs for the matrices were set to different values (1.0, 0.96, 0.93, 0.98, and 0.995, respectively). The search profile differentiated clearly between the sets of regulated genes and those whose expression was unchanged in lung tumors of c-Myc transgenic mice.

#### Gene reporter assays

A detailed description of the development of a dual Luciferase gene reporter assay is given in our previous works [6, 7]. Essentially, 2 Kb regulatory regions centered on the Transcription Start Site (TSS) of the *Adam19, Traf4,* and *Map2k1* genes were PCR amplified from mouse genomic DNA (primer sequences used for cloning are listed in Supplementary Table 5) and inserted into pCR4-TOPO shuttle vector through TOPO-TA cloning (Invitrogen). Promoter fragments were further cloned into our destination pCZ-REN_LUC retroviral dual reporter vector using classical restriction sites, such as Bam HI/Hind III for Adam19 and Map2k1 [6] or Sfi I/Pac I (for Traf4, using a modified version of pCZ-REN_LUC reporter with a linker region containing the additional cloning sites). HEK 293T cells were transfected with 350 ng of the reporter vectors along with 250 ng of the expression vector (empty or over-expressing c-Myc) using Lipofectamine 2000 according to the manufacturer's recommendations (Invitrogen). The c-Myc over-expressing vector MIG-MYC was used to examine the responsiveness of the promoters at low and high c-Myc protein level. Dual luciferase assays were done 24hrs after transfection using commercial reagents (Promega) and a multi-plate reader (Victor 3, Perkin Elmer).

#### Chromatin immuno precipitation (ChIP) assays

ChIP assays were performed on human HEK 293T cells upon transient over-expression of c-Myc. The transfection conditions, Chromatin IP protocol, and qPCR measurements were done as recently reported [6, 7]. Specifically, primer pairs for the promoter regions of *ADAM19, MAP2K1,* and *TRAF4* genes were designed within the locations of positive signals of publically available ChIP-seq data (see below) using the Primer 3 online tool (http://bioinfo.ut.ee/primer3/). Genomic regions in the genes *ATCB* and *CCNB2* served as negative control. Sequences of primers used for ChIP-qPCR are shown in Supplementary Table 5

#### ChIP-seq data retrieved from the UCSC Genome Browser

c-Myc ChIP-seq data deposited in the UCSC Genome Browser (http://genome.ucsc.edu/) was retrieved from the ENCyclopedia Of DNA Elements (ENCODE) consortium (hg19-human or mm9-mouse genomic data) as previously described [7]. Data of 7 human cell lines derived from 8 different experiments and 2 mouse cell lines were analyzed for c-Myc binding sites. The findings were compared with orthologue gene promoters and other genomic sequences of differentially expressed genes determined for lung tumors of c-Myc transgenic mice. As shown in Supplementary Table 3 ChIP-seq data confirmed c-Myc binding for 76% and 81% of up-and down-regulated genes, respectively.

#### Kaplan–Meier survival statistics

Kaplan–Meier curves for significantly regulated genes in c-Myc transgenic tumors were generated with the online tool Kaplan–Meier Plotter (http://kmplot.com/analysis/) [80]. A total of 720 whole genome data sets of lung adeno- and squamous cell carcinoma patients were interrogated and 60 out of 100 regulated genes (14 up- and 46 down-regulated) were significantly associated with survival (Supplementary Table 4). We also generated a gene signature, viz. “13-gene signature” by combining the up-regulated genes over-expressed in murine lung tumors. The combined HR was calculated with Kaplan–Meier Plotter tool by considering the mean expression levels of selected genes. The patients were split in two groups according to the expression levels (high vs. low) and survival for up to 20 years was determined. Note, c-Myc itself is excluded in this signature. Furthermore, a classifier signature was established based on 16 genes that showed the strongest regulation (the average FC in the small, middle and large c-Myc transgenic tumors were about 10-fold) and were significantly associated with poor prognosis when expressed at low levels, i.e. the “16-gene signature” (HR were calculated with the same approach as described above).

#### Clinical significance and druggability of newly identified c-Myc target genes

To further determine the clinical relevance of differentially expressed genes in response to c-Myc over-expression two independent publically available datasets for mRNA and/or protein expression were interrogated. Specifically, RNA-seq data of human lung adenocarcinoma generated with the Illumina Hiseq platform by the TCGA Consortium (The Cancer Genome Atlas, https://cancergenome.nih.gov/) were interrogated. The data were downloaded from TCGA_Pancancer datasets using the Synapse online tool (https://www.synapse.org/; Synapse ID: syn418003; [81]) and 57 normal tissues and 355 tumor tissues from lung adenocarcinoma patient samples were analyzed. Altogether, expression values were collected, scatter plots prepared and statistical analysis was performed. The same approach was utilized to analyze data of lung squamous cell carcinomas. For this purpose expression values from 17 normal tissues and 220 tumor tissues from lung squamous cell carcinoma-derived data sets were retrieved (Synapse ID: syn1461168; [82]). Demographic information of the analyzed cohorts (both affected by lung adenocarcinoma and squamous cell carcinoma) is given in Supplementary Table 7. Additionally, protein expression of the newly identified c-Myc target genes (Supplementary Table 4) was assessed by immunohistochemistry using information deposited in The Human Protein Atlas online resource for lung cancer tissues (http://www.proteinatlas.org/cancer). Typically, 9 to 12 human-derived lung cancer patients were considered and the number of patients with high (H, combining high and medium signals) or low (L, combining low with absent signals) expression of the protein was calculated (Supplementary Table 4).

## SUPPLEMENTARY MATERIALS FIGURES AND TABLES














