# Correction: Oncogenomics of c-Myc transgenic mice reveal novel regulators of extracellular signaling, angiogenesis and invasion with clinical significance for human lung adenocarcinoma

**DOI:** 10.18632/oncotarget.26489

**Published:** 2018-12-14

**Authors:** Yari Ciribilli, Jürgen Borlak

**Affiliations:** ^1^ Centre for Integrative Biology (CIBIO), University of Trento, 38123 Povo (TN), Italy; ^2^ Centre for Pharmacology and Toxicology, Hannover Medical School, 30625 Hannover, Germany

**This article has been corrected:** In the Materials and Methods section, regarding the Isolation of RNA, production of cRNA, array hybridization and scanning, the correct microarray GEO information is given below:

**Isolation of RNA, production of cRNA, array hybridization and scanning**

Total RNA was extracted using the Qiagen RNA isolation kit according to the manufacturer’s instructions (Qiagen, Hilden, Germany) and copy RNA was prepared according to the Affymetrix Gene Chipâ Expression Analysis Technical Manual (Santa Clara, CA). Further details are given in our recent report [6]. The microarray study is MIAME compliant, and the raw data have been deposited in GEO database (GSE54829; http://www.ncbi.nlm.nih.gov/geo).

Original article: Oncotarget. 2017; 8:101808-101831. https://doi.org/10.18632/oncotarget.21981

