# Supplementary material for: Oncogenomics of c-Myc transgenic mice reveal novel regulators of extracellular signaling, angiogenesis and invasion with clinical significance for human lung adenocarcinoma
Source: Oncotarget. 2017 Oct 23;8(60):101808–31. doi: 10.18632/oncotarget.21981 (PMC5731916; doi:10.18632/oncotarget.21981)
Supplement: Supplementary file 1 [file oncotarget-08-101808-s001.pdf]

Oncogenomics of c-Myc transgenic mice reveal novel regulators of extracellular signaling, angiogenesis and invasion with clinical significance for human lung adenocarcinoma

SUPPLEMENTARY MATERIALS

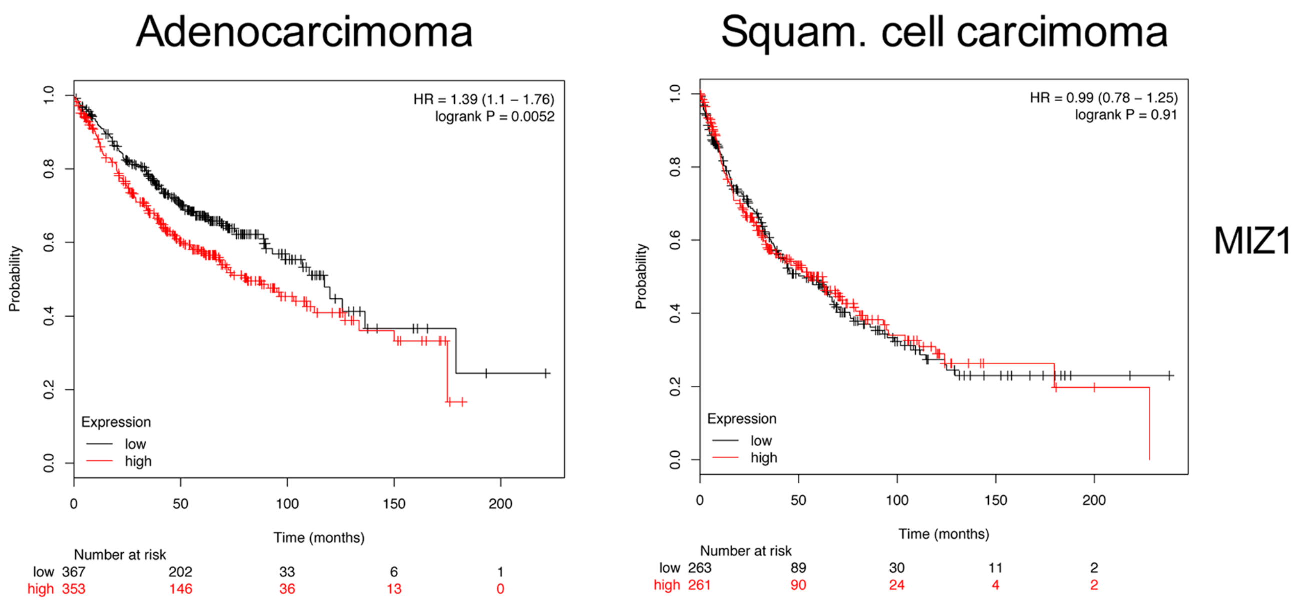

Supplementary Figure 1: Kaplan–Meier survival statistics of Miz1. Kaplan–Meier curves of lung adenocarcinoma (**left panel**) and lung squamous cell carcinoma (**right panel**) were generated using KM plotter tool by considering the expression of the c-Myc cofactor Miz1. Red and black curves always represent patients with high and low expression levels of Miz1. Given underneath each curve is the number of patients considered. Additionally the Hazard Ratios and the *p*-values are shown in the plot.

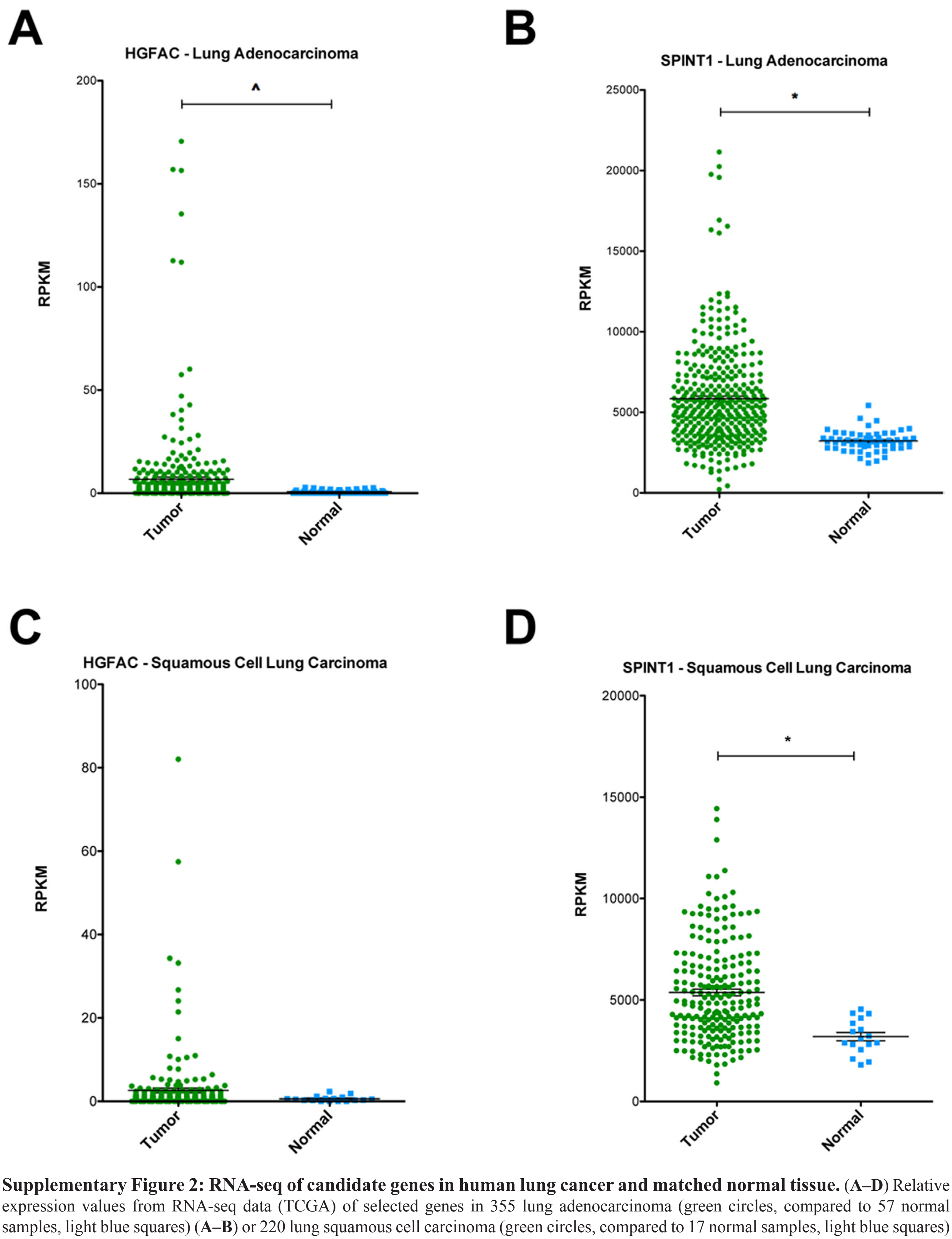

Supplementary Figure 2: RNA-seq of candidate genes in human lung cancer and matched normal tissue. (**A–D**) Relative expression values from RNA-seq data (TCGA) of selected genes in 355 lung adenocarcinoma (green circles, compared to 57 normal samples, light blue squares) (**A–B**) or 220 lung squamous cell carcinoma (green circles, compared to 17 normal samples, light blue squares) (**C–D**). Shown are the means and standard errors. ^ = $p < 0.05$; * = $p < 0.01$.

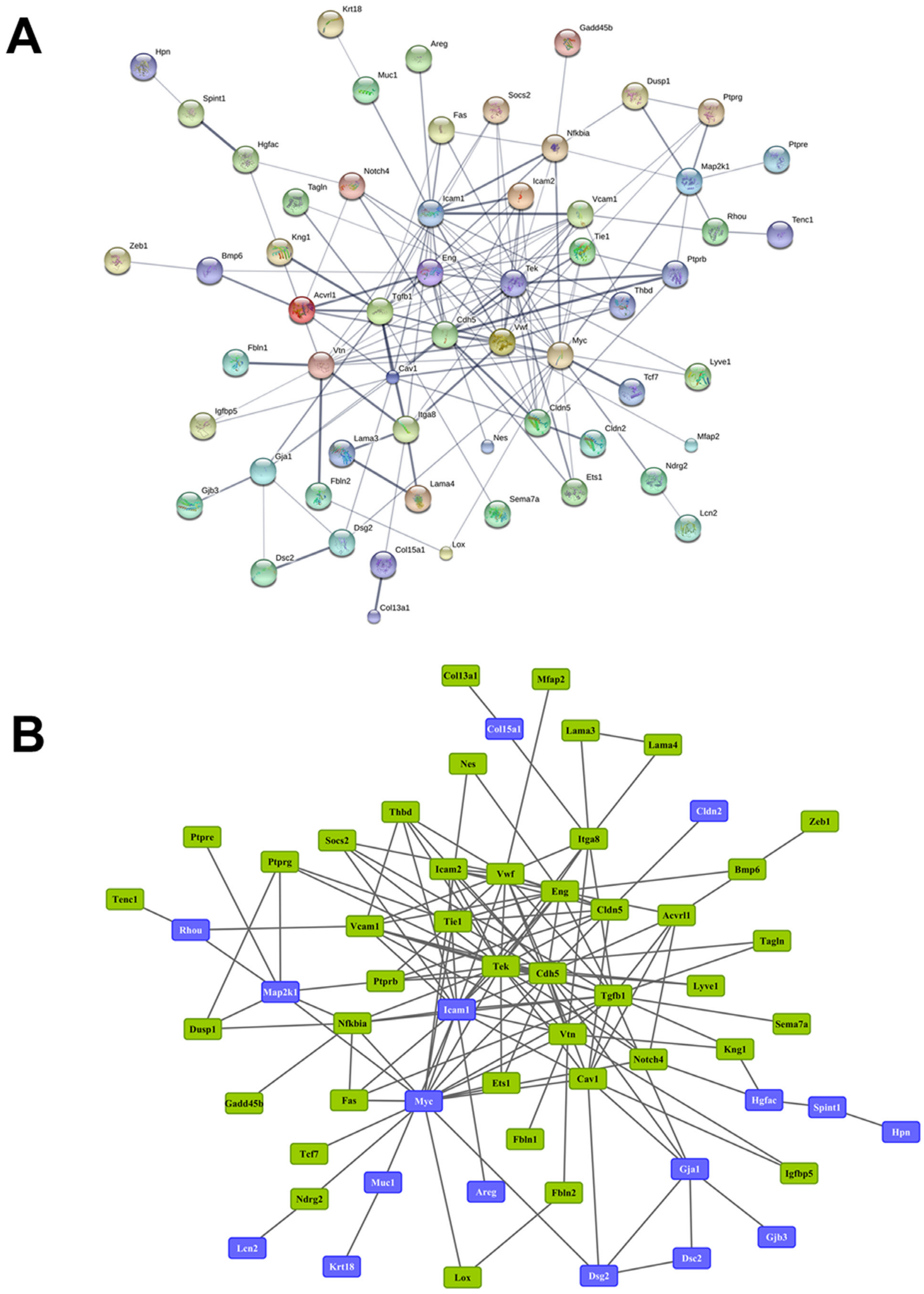

Supplementary Figure 3: STRING protein-protein interaction network of c-Myc regulated genes in papillary lung adenocarcinomas. A total of 59 out of 97 DEGs interact with each. Shown in panel A is the String PPI network. Shown in panel B is the String PPI network visualized by Cytoscape (the blue and green color nodes are up- and down regulated DEGs, respectively).

Supplementary Table 1: Comprehensive listing of significantly regulated genes in c-Myc-induced lung papillary adenocarcinomas. See Supplementary_Table_1

Supplementary Table 2: Fold changes of gene expression in lung tumors determined by RT-PCR and microarray analysis

Gene	Method	Mean FC ± SD		
		Size of tumors		
		Small	Medium	Large
Ros1	RT-PCR	3.21 ± 0.73	3.07 ± 1.02	3.17 ± 0.19
	Affymetrix	9.26 ± 2.23	13.22 ± 4.41	21.85 ± 4.77
Ect2	RT-PCR	3.14 ± 1.25	3.18 ± 0.90	3.50 ± 1.31
	Affymetrix	10.25 ± 1.34	4.61 ± 1.80	4.44 ± 2.06
Traf4	RT-PCR	2.58 ± 0.40	2.56 ± 0.40	2.74 ± 0.42
	Affymetrix	4.30 ± 0.41	5.02 ± 0.50	4.49 ± 0.22
Hgfac	RT-PCR	3.32 ± 0.15	4.77 ± 0.50	5.11 ± 0.87
	Affymetrix	4.70 ± 0.03	9.57 ± 0.59	8.65 ± 0.43
Adam19	RT-PCR	2.98 ± 0.99	3.74 ± 0.44	3.84 ± 0.23
	Affymetrix	3.17 ± 0.24	3.33 ± 1.31	3.14 ± 0.70
Bmp6	RT-PCR	–7.28 ± 2.49	–11.86 ± 2.91	–33.03 ± 7.14
	Affymetrix	–15.47 ± 1.26	–34.84 ± 0.88	–22.81 ± 1.05
Acvrl1	RT-PCR	–6.86 ± 1.65	–18.83 ± 4.92	–22.59 ± 7.00
	Affymetrix	–5.89 ± 1.02	–6.73 ± 0.79	–7.59 ± 0.45
Igfbp5	RT-PCR	–3.18 ± 1.03	–3.05 ± 0.73	–2.81 ± 1.05
	Affymetrix	–6.51 ± 0.73	–1.94 ± 1.43	–4.85 ± 1.38
Sema3f	RT-PCR	–2.27 ± 0.13	–3.25 ± 1.13	–3.48 ± 1.10
	Affymetrix	–4.90 ± 0.28	–5.40 ± 0.41	–5.60 ± 0.29
Cav1	RT-PCR	–2.91 ± 0.94	–4.88 ± 0.46	–6.19 ± 2.17
	Affymetrix	–4.04 ± 1.03	–5.64 ± 2.24	–6.54 ± 3.17
Cdh5	RT-PCR	–2.17 ± 0.27	–2.87 ± 0.22	–3.30 ± 0.52
	Affymetrix	–7.18 ± 1.62	–9.90 ± 1.04	–9.95 ± 1.68

Supplementary Table 3: Summary of ENCODE ChIP-seq data from human and murine databases. See Supplementary_Table_3

Supplementary Table 4: Clinical relevance of c-Myc responsive genes. See Supplementary_Table_4

Supplementary Table 5: Primer sequences used for RT-PCR, RT-qPCR, ChIP-qPCR and cloning experiments. See Supplementary_Table_5

Supplementary Table 6: Sequences of oligonucleotide probes for EMSA assays

Mouse_Map2k1	5′-GGTTCTCCGCGTGGGTTGGGCGG
Mouse_Rhou	5′-CGGGGTCCGCGTGGGTTGGCGTG
Mouse_Traf4	5′-TGAGCGGCGCGTGGCGCAGGAGA
Mouse_Gjb3	5′-GTGAGCCACCACGTGGTTGCTAG
Mouse_Gja1	5′-GTTGACTTCCACGTGGTTCTCTC
Mouse_Dsg2	5′-TGAGAAGCCCCACGCGCACCCTC
Mouse_Adam19	5′-CCTCCTCCGCGTGGCGCCTCCCC
Mouse_Fas	5′-GTGACATACACGTGTTCAAAGCG
Mouse_Hoxa5	5′-CAACAAAAGCACGTGATTCGAAG
Mouse_Acvrl1	5′-CAGACGGCGCGTGGCAGGGTCAG
Mouse_Akap12	5′-CAGCTCCTGCCACGTGAGCCGCC
Mouse_Vcam1	5′-TTTCTCTCACGTGGCACTTATGA
Mouse_Meox2	5′-ATTCAGGAGCACGTGTTTCACTG
Mouse_Sema7a	5′-TTGCCCGCACGTGGCAGTTGTCA
Mouse_Sema3c	5′-TGCGGCGGGCCACGCGGGGCCGC
c-Myc_positive control _WT	5′-GGAAGCAGACCACGTGGTCTGCTTCC
c-Myc_positive control_ Mut	5′-GGAAGCAGACCACGGAGTCTGCTTCC

Supplementary Table 7: Demographic information of (A) lung adenocarcinoma and (B) squamous cell carcinoma patients from the TCGA consortium database. See Supplementary_Table_7
